# Negative effects of long-term feeding of high-grain diets to lactating goats on milk fat production and composition by regulating gene expression and DNA methylation in the mammary gland

**DOI:** 10.1186/s40104-017-0204-2

**Published:** 2017-10-01

**Authors:** Ping Tian, Yanwen Luo, Xian Li, Jing Tian, Shiyu Tao, Canfeng Hua, Yali Geng, Yingdong Ni, Ruqian Zhao

**Affiliations:** 1 Key Laboratory of Animal Physiology & Biochemistry, Nanjing Agricultural University, Nanjing, 210095 People’s Republic of China; 2 College of Veterinary Medicine, Northwest A and F University, Yangling, Shannxi China

**Keywords:** DNA methylation, Gene expression, Goat, High concentrate diet, Milk fat

## Abstract

**Background:**

It is well known that feeding a high concentrate (HC) diet to lactating ruminants likely induces subacute ruminal acidosis (SARA) and leads to a decrease in milk fat production. However, the effects of feeding a HC diet for long periods on milk fatty acids composition and the mechanism behind the decline of milk fat still remains poorly understood. The aim of this study was to investigate the impact of feeding a HC diet to lactating dairy goats on milk fat yield and fatty acids composition with an emphasis on the mechanisms underlying the milk fat depression. Seventeen mid-lactating dairy goats were randomly allocated to three groups. The control treatment was fed a low-concentrate diet (35% concentrate, *n* = 5, LC) and there were two high-concentrate treatments (65% concentrate, HC), one fed a high concentrate diet for a long period (19 wks, *n* = 7, HL); one fed a high concentrate diet for a short period of time (4 wk, *n* = 5, HS). Milk fat production and fatty acids profiles were measured. In order to investigate the mechanisms underlying the changes in milk fat production and composition, the gene expression involved in lipid metabolism and DNA methylation in the mammary gland were also analyzed.

**Results:**

Milk production was increased by feeding the HC diet in the HS and HL groups compared with the LC diet (*P* < 0.01), while the percentage of milk fat was lower in the HL (*P* < 0.05) but not in the HS group. The total amount of saturated fatty acids (SFA) in the milk was not changed by feeding the HC diet, whereas the levels of unsaturated fatty acids (UFA) and monounsaturated fatty acids (MUFA) were markedly decreased in the HL group compared with the LC group (*P* < 0.05). Among these fatty acids, the concentrations of C15:0 (*P* < 0.01), C17:0 (*P* < 0.01), C17:1 (*P* < 0.01), C18:1n-9c (*P* < 0.05), C18:3n-3r (*P* < 0.01) and C20:0 (*P* < 0.01) were markedly lower in the HL group, and the concentrations of C20:0 (*P* < 0.05) and C18:3n-3r (*P* < 0.01) were lower in the HS group compared with the LC group. However, the concentrations of C18:2n-6c (*P* < 0.05) and C20:4n-6 (*P* < 0.05) in the milk fat were higher in the HS group. Real-time PCR results showed that the mRNA expression of the genes involved in milk fat production in the mammary gland was generally decreased in the HL and HS groups compared with the LC group. Among these genes, *ACSL1*, *ACSS1* & *2*, *ACACA*, *FAS*, *SCD*, *FADS2,* and *SREBP1* were down-regulated in the mammary gland of the HL group (*P* < 0.05), and the expressions of *ACSS2*, *ACACA,* and *FADS2* mRNA were markedly decreased in the HS goats compared with the LC group (*P* < 0.05). In contrast to the gene expression, the level of DNA methylation in the promoter regions of the *ACACA* and *SCD* genes was increased in the HL group compared with the LC group (*P* < 0.05). The levels of ACSL1 protein expression and FAS enzyme activity were also decreased in the mammary gland of the HL compared with the LC group (*P* < 0.05).

**Conclusions:**

Long-term feeding of a HC diet to lactating goats induced milk fat depression and FAs profile shift with lower MUFAs but higher SFAs. A general down-regulation of the gene expression involved in the milk fat production and a higher DNA methylation in the mammary gland may contribute to the decrease in milk fat production in goats fed a HC diet for long time periods.

**Electronic supplementary material:**

The online version of this article (doi:10.1186/s40104-017-0204-2) contains supplementary material, which is available to authorized users.

## Background

Milk contains high levels of nutrients such as proteins, fatty acids, phospholipids, vitamins and minerals [1]. Among these nutrients, milk fat plays an important role in determining the quality and energy composition of dairy products [2]. Milk fat contains large amount of saturated fatty acids (SFAs) and unsaturated fatty acids (UFAs). Recently, evidence has shown that high levels of SFAs pose a potential risk to human health such as cardiovascular disease (CVD) [3]. In contrast, there is epidemiological evidence suggesting that dietary monounsaturated fatty acids (MUFAs) and polyunsaturated fatty acids (PUFAs) have beneficial effects for preventing CVD by favorably affecting a number of risk factors for CVD, including plasma lipids and lipoproteins. For example, oleic acid has a protective effect against retinopathy [4]. Similarly, eico-sapentaenoic acid (EPA) and docosahexaenoic acid (DHA) play beneficial roles in preventing diabetes, atherosclerosis and arthritis [5]. Many strategies have been investigated to enhance the unsaturated fatty acid content of milk [6–8].

Milk triglycerides are derived from two sources: the biosynthesis of fatty acids and their subsequent esterification within the mammary gland and the uptake of lipids from plasma into the mammary gland [9]. Fatty acids in the mammary gland can also be generated by two pathways: short- and medium-chain fatty acids (C4-C14) can be synthesized de novo under the control of several key factors and enzymes including SREBP-1 (sterol regulatory element binding protein 1), ACACA (acetyl-coenzyme A carboxylase alpha), FAS (fatty acid synthase), and ACSS2 (acyl-CoA synthetase short-chain family member 2). Long-chain fatty acids (C > 16) are taken up from plasma via transporters such as fatty acid binding protein (FABP), fatty acid transport protein (FATP) and cluster of differentiation 36 (CD36) [10]. Acetate and 3-hydroxybutyrate are the main sources of a de novo synthesis for short-and medium-chain FAs [11]. These FAs in the mammary gland can be desaturated by stearoyl-CoA desaturase (SCD) and a synthesis of cis-9 unsaturated FA and can then be esterified to glycerol sequentially via glycerol-3phosphate acyl transferase (GPAT), acyl glycerol phosphate acyl transferase (AGPAT), and diacylglycerol acyltransferase (DGAT) [11]; subsequently, the triglycerides are secreted into the milk as fat globules. The composition of milk fatty acids is highly variable and depends on several factors such as the animal breed, the lactating stage, the dietary lipids composition, the energy status, and the feeding regimes [12–14]. High levels of FAs are mobilized from adipose tissue in early lactation to provide more nutrients for the milk synthesis [12, 13]. In adipose tissue, fatty acids of 18:1c9, 16:0, and 18:0 account for nearly 90% of total FAs [14] that are released into the blood during the negative energy balance of early lactation. These FAs are taken up through blood into the mammary gland and ultimately inhibit the de novo synthesis of fatty acids [13, 15]. As a consequence, the concentration of short- and medium-chain FAs in milk is lower during the early lactation than the later lactation [13, 16]. However, it has been reported that during the early lactation, unsaturated fatty acids such as conjugated linoleic acid, linoleic acid, and omega-3 were enriched in the milk [17, 18].

Most dairy animals in intensive production systems are fed high levels of grain to maximize energy intake and milk production. However, excessive amounts of non-structural carbohydrates and highly fermentable forage will lead to a rapid fermentation and the accumulation of organic acids in the rumen [19], which likely induces subacute ruminal acidosis (SARA) [19, 20]. Both acute and subacute ruminal acidosis can decrease the production of milk fat and cause milk fat depression and the shift of fatty acids profiles in lactating cows [20]. To our knowledge, the effects of long-term (more than 4 mo) feeding of a high concentrate (HC) diet to lactating ruminants on the production and composition of milk fat and the relevant mechanisms behind milk fat alterations are still unknown. In this study, mid-lactating dairy goats were fed a HC diet for a long (19 wk) or short (4 wk) period. Milk fat and FAs profiles were measured and the relevant gene expression and DNA methylation in the mammary gland were evaluated to investigate the mechanisms underlying the changes in milk fat production and composition.

## Methods

### Animals and experimental procedures

**Fig. 1:**
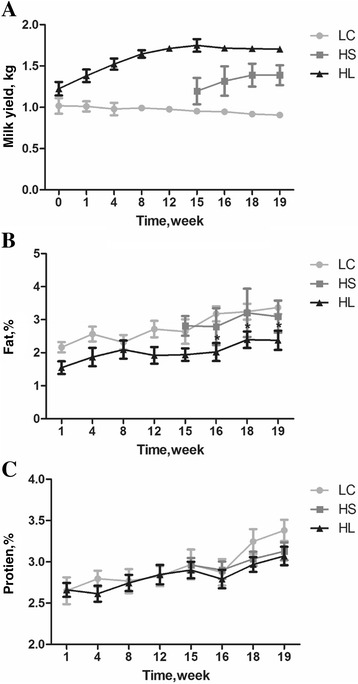
The changes of milk yield, milk fat and protein. **a** Daily milk yield (kg/d); **b** Milk fat content (%); **c** Milk protein content (%). The asterisk * indicates a significant difference between HL and LC control group

Seventeen healthy, mid-lactating goats (Guanzhong dairy goats, 60 ± 5 d of lactation) with an average initial body weight of 49.7 ± 5.5 kg (mean ± SD) and similar daily milk yield (1.18 ± 0.13 kg/d) were selected and housed individually (square measure: 3.0 ~ 3.2 m^2^) in a standard animal feeding house at Northwest A and F University (Shanxi, China). Prior to the experiment, all goats were allowed free access to a control diet containing a forage to concentrate ratio of 65:35 for 2 wk. Ingredients and chemical composition of the experimental diets were shown in the Additional file 1: Table S1. After dietary adaptation, goats were randomly assigned to three groups, and the daily milk yield in each group prior to the start of experiment did not show a significant difference as shown in Fig. 1 (*P* > 0.05). The control treatment was fed a low-concentrate diet (35%concentrate, *n* = 5, LC) while there were two high concentrate treatments (65% concentrate, HC), one fed a high concentrate diet for a long period (19 wk, *n* = 7, HL), and the other fed a high concentrate diet for a short period of time (4 wk, *n* = 5, HS) after 15 wk of low concentrate diet. All goats were fed daily at 08:00 and 18:00, respectively, and milked twice daily before feeding.

### Sample collection and assay

At the end of the experiment, goats were euthanized after overnight fasting. All goats were killed with neck vein injections of xylazine [0.5 mg/kg body weight; Xylosol; Ogris Pharme, Wels, Austria] and pentobarbital [50 mg/kg body weight; Release; WDT, Garbsen, Germany]. After euthanasia, a portion of mammary gland tissues were collected and immediately frozen in liquid nitrogen, and then used for total RNA, genomic DNA and protein extraction.

### Milk fatty acid analysis

Milk samples were collected twice daily before feeding at 08:00 and 18:00, respectively. The samples of milk were mixed thoroughly, and one portion was stored at 4 °C for milk fat and protein analysis, another was stored at −70 °C for fatty acids composition analysis. The level of milk fatty acids was detected following the standard protocol of gas chromatography in Jiangsu Academy of Agricultural Sciences as previously described [21].

### RNA extraction and real-time quantitative PCR

Total RNA was extracted from each dairy goat using TRIZOL reagent (Takara, Dalian, China) according to the manufacturer’s protocol, and total RNA concentration was then quantified by measuring the absorbance at 260 nm in a NanoDropND-1000 Spectrophotometer (Thermo Fisher Scientific, Madison, WI, USA). The quality of total RNA was also verified by electrophoresis. Then two micrograms of total RNA were treated with RNAse-Free DNase and reverse transcribed according to manufacturer’s instructions (Takara, Dalian, China). The qRT-PCR efficiency was determined with serial 4-fold cDNA dilutions (1, 1/4, 1/16, 1/64, 1/256 cDNA template), and the efficiency was high with values between 95 and 100%. Two microliter of diluted cDNA (1:16 vol/vol) was used for real-time PCR which was performed in Stratagene Mx3000P qPCR instrument (Agilent, California, USA). The information of primer sequence was shown in Table 1. The mRNA expression of *18S rRNA*, *β-actin* and *GAPDH* genes was measured in the mammary gland tissues by real-time PCR, and *GAPDH* was finally used as a reference gene for normalization purpose due to the high efficiency and the stable expression in all samples. The method of 2^-△△Ct^ was used to analyze the real-time PCR results and gene mRNA levels were expressed as the fold change relative to the mean value of control group [22].

**Table 1:** PCR primer sequences of the target genes

Target genes	Primer sequences	GenBank accession No.	PCR products, bp
*LPL*	F:5′-CAAGTCGCCTTTCTCCCGAT-3’	NM_001285607.1	177
	R:5′-CTGCAATCACACGGAGAGCTT-3’		
*ACACA*	F:5′-TATGACGGCAGCAGTTACACC-3’	XM_018064168.1	201
	R:5′-CACCTCGATCTCAGCATAGCAC-3’		
*FAS*	F:5′-CCCCAAGCTCTTTGACAACCG-3’	NM_001285629.1	100
	R:5′-CAGCTCCTTGTACACGTCACC-3’		
*FABP3*	F:5′-TGAGACCACGGCAGATGA-3’	NM_001285701.1	230
	R:5′-TAGCCCACTGGCAGAAGA-3’		
*FABP4*	F:5′-CATAAACTTAGATGAAGGTGCTC-3’	NM_001285623.1	116
	R:5′-CACCGTTCATGACACATTCCA −3’		
*DGAT1*	F:5′-AAGCCCTTCAAGGACATG-3’	XM_018058728.1	100
	R:5′-AGAGCCAGTAGAAGAAGATG-3’		
*CPT1B*	F:5′- CCAGCCACAGTTCATCGGTA-3’	XM_018048994.1	103
	R:5′-CAGTCTTCTCCTCGAACTCC-3’		
*ACSS1*	F:5′-TGAGCGACTGCGACTTCC-3’	XM_018057282.1	223
	R:5′-CGGCGGACTCCATACCTCT-3’		
*ACSS2*	F:5′-TGAGCCAGAGGAAACCA-3’	XM_005688483.3	157
	R:5′-ACACGCCAGCATAGCC-3’		
*ACSL1*	F:5′-CGCAGTGGCATCATTAG-3’	XM_018041881.1	183
	R:5′-CTCGGTCTGTCCGTAGC-3’		
*SREBP1*	F:5′-ACGCCATCGAGAAACGCTAC-3’	NM_001285755.1	181
	R:5′-GTGCGCAGACTCAGGTTCTC-3’		
*PPARγ*	F:5′-CCTTCACCACCGTTGACTTCT-3’	NM_001285658.1	145
	F:5′-GATACAGGCTCCACTTTGATTGC-3’		
*CD36*	F:5′-ATAATACTGCGGATGGA-3’	NM_001285578.1	165
	R:5′-TCTCAACGAAAGGTGG-3’		
*LDLR*	F:5′-GTGGCTGACACCAAAGGG-3’	XM_005682375.3	175
	R:5′-CGGTCACCAGCGAGTAAAT-3’		
*SCD*	F:5′-GTGGGTTGGCTGCTTG-3’	NM_001285619.1	243
	R:5′-CCAGGTGGCATTGAGC-3’		
*FADS2*	F:5′-GCATCGCCTGGTTCACT-3’	XM_018043056.1	249
	R:5′-GGAAGATGTTGGGTTTGG-3’		
*GAPDH*	F:5′-GGGTCATCTCTCTGCACCT-3’	XM_005680968.3	176
	R:5′-GGTCATAAGTCCCTCCACGA-3’		

### Western blotting analysis

Frozen mammary gland (100 mg) was minced and homogenized in 1 mL of ice-cold RIPA buffer (pH 8.0, 50 mmol/L Tris, 150 mmol/L NaCl, 1.0% Triton X-100, 0.5% sodium deoxycholate, 0.1% SDS) containing a protease inhibitor cocktail (EDTA-free; 50 × Conc.) (Roche Applied Science, Penz-berg, Germany). Then the homogenates was centrifuged for 20 min at 12,000×g at 4 °C. Protein concentrations were measured with a Pierce BCA Protein Assay Kit (No.23225, Thermo, USA). Sixty micrograms of protein extract from each sample were separated by electrophoresis in 7.5% or 10% SDS-PAGE, transferred onto nitrocellulose membrane (BioTrace, Pall Co, USA). After transferred, membranes were blocked for 2 h at room temperature in blocking buffer (3% albumin from bovine serum), then incubated overnight at 4 °C with the following primary antibodies: rabbit anti-mouse α-Tublin (Bioworld, BS1699 1:500), goat anti-mouse ACSL1 (santa cruz, sc-98,925, 1:200), goat anti-human SCD (santa cruz sc-23,016, 1:200). In western blot detection, α-tublin was used as the internal control. Then the blots were incubated with the rabbit anti-goat horseradish peroxidase (HRP)-conjugated second antibody (E030130–01, Earth Ox, CA, 1:10,000) or goat anti-rabbit HRP-conjugated second antibody (Bioworld, BS13278, 1: 10,000) for 2 h at 25 °C, and the bound HRP activity was detected by use of VersaDoc Imaging System (Bio-Rad, CA, USA).

### DNA methylation assay

Genomic DNA was extracted from mammary gland tissues using a commercial kit (DP304, TIANGEN Biotech Co., LTD. Beijing, China). In brief, 100 mg mammary gland powder was incubated with 1 mL lysis buffer (pH 8.0, 50 mmol/L Tris; 100 mmol/L EDTA; 100 mmol/L NaCl; 1.0% SDS) containing phenol, chloroform and 50 μL proteinase K (10 mg/mL stock) at 55 °C for 2 h, and then centrifugated at 12,000×g for 5 min. About 500 μL supernatant was collected, and incubated with 500 μL isopropyl alcohol and 60 μL 3.0 mol/L sodium acetate (pH 5.2) at room temperature for 5 min, and then centrifugated at 12,000 rpm for 15 min. The pellet was washed twice with 70% ethanol, after drying the pellet was finally resuspended in TE buffer (pH 8.0, 10 mmol/L Tris, 1 mmol/L EDTA). Samples were incubated at 65 °C in a shaking water bath for 1 h to ensure a good resuspension.

The isolated genomic DNA was sonicated to produce random fragments with the size from 300 to 500 bp. The sonication condition was set up with the following parameters: output power 30 W, 5 s pulse on and 5 s pulse off, 10 cycles. Two microgram of sonicated genomic DNA was heat-denatured to produce single-stranded DNA, and the same portion of the un-denatured DNA was stored as control (input) DNA. The methylated DNA fragments was immune-precipitated by the mouse monoclonal antibody against 5-methyl cytidine (ab10805, Abcam). Precleared Protein A/G Plus Agarose (Santa Cruz, Dallas, TX) was used to immune-precipitate the antibody/DNA complexes, and the MeDIP DNA was purified. Forty nanogram of MeDIP DNA and control input DNA was used to amplify the *ACACA*, *SCD*, *FAS*, *ACSL1*, *ACSS1* & *2*, *FADS2* promotor regions by real-time PCR with specific primers (Table 2). The primers were designed with the software of “Methyl Primer Express” using the specific promotor sequence enriched with CpG sites blasted from the website of “http://www.ensembl.org/index.html”. The ratios of the signals in the immunoprecipitated DNA vs input DNA were calculated as a measure for representing the relative enrichment of methylation in the particular sample.

**Table 2:** PCR primer sequences using for MeDIP

Target genes	MeDIP primer sequence	GenBank accession No.	PCR products, bp
*FADS2*	F:5′-GGCACTAATCCCAAGCAG-3’	XM_018043056.1	138
	R:5′-GGAAACAATACAGGACCTCATA-3’		
*ACSL1*	F:5′-GGGAGGCGAGCAGAAAGA-3’	XM_018041881.1	142
	R:5′-AACAGACGGTGGAGGGTG-3’		
*ACSS1*	F:5′-ACCTGAGAAGGGATGTGG-3’	XM_018057282.1	104
	R:5′-AGAAGACTCAACGCAAACA-3’		
*FAS*	F:5′-TTGCCTAAAGTCAGTGTCG-3’	NM_001285629.1	169
	R:5′-GCAGGTCAACCGCATAAC-3’		
*ACACA*	F:5′-GCTTTCTTCACCGAGGCT-3’	XM_018064168.1	173
	R:5′-CGGAGGGTATCGCATTCA-3’		
*ACSS2*	F:5′-CCTCCCGTTCTGCTTTCC-3’	XM_005688483.3	132
	R:5′-AGCCGTGCCTGGTGGTGTTG-3’		
*SCD*	F:5′-CCCCAGTGCCCATCCATTT-3’	NM_001285619.1	168
	R:5′-TCCCTTTCTCCTCGGCTTCTC-3’		

### FAS enzyme activity assay

FAS enzyme activity in mammary gland tissues was measured by FAS activity Assay Kit (jiancheng Bioengineering Institute, Nanjing, China).

### Statistical analysis

All data are presented as the mean ± SEM. The data of milk yield and milk composition was analyzed for differences due to diet treatment, time effect, and their interaction by using PROC MIXED, SAS 9.3, (SAS Institute Inc., Cary, NC, USA). The data of milk yield, milk fat and protein obtained before the beginning of the treatment was considered as a co-variable in the statistical analysis. The differences of parameters in fatty acids composition, gene and protein expression, enzyme activities in mammary gland were analyzed by using the post hoc analysis with the least significant difference test following ANOVA of SPSS 11.0. The 2^-ΔΔCt^ method was applied to analyze the real-time PCR data. Differences were considered significant at *P* < 0.05, and 0.05 < *P* < 0.1 is considered as a tendency. Numbers of replicates used for statistics are noted in the Tables and Figures.

## Results

### Milk yield and production of milk protein and fat

As shown in Fig. 1a-c, goats fed a high concentrate diet (HL and HS group) produced (*P* < 0.01) more milk than goats fed a low concentrate diet (LC). Diet significantly affected the milk fat production (*P* < 0.05). The percentage of milk fat decreased in the HL group after 16 wk and was significantly different from the control group (LC) (*P* < 0.05), while the protein was not significantly affected by dietary treatment.

### Milk fatty acid composition

The composition of the milk fatty acids was analyzed and is shown in Table 3. Compared with the LC group, the HL group exhibited a lower level of unsaturated fatty acids (UFA) and monounsaturated fatty acids (MUFA) in the milk (*P* < 0.05), while there was no significant difference between the LC and HS groups (*P* > 0.05). The content of saturated fatty acids (SFA) in the milk showed a tendency to increase in the HL group compared with the LC group (0.05 < *P* < 0.1). Compared with LC, the concentration of C15:0 (*P* < 0.01), C17:0 (*P* < 0.01), C17:1 (*P* < 0.01), C18:1n-9c (*P* < 0.05), C20:0 (*P* < 0.01) and C18:3n-3r (*P* < 0.01) in the milk was markedly lower in the HL group and the concentrations of C20:0 (*P* < 0.05) and C18:3n-3r (*P* < 0.01) were lower in the HS group. Moreover, the contents of C18:2n-6c (*P* < 0.05) and C20:4n-6 (*P* < 0.05) in the milk were higher in the HS compared with the LC group.

**Table 3:** Fatty acids composition in the milk fat

Fatty acids	LC	HS	HL
C4:0	0.78 ± 0.05	0.79 ± 0.06	0.91 ± 0.08
C6:0	1.19 ± 0.06	1.16 ± 0.08	1.27 ± 0.11
C8:0	1.45 ± 0.08	1.38 ± 0.10	1.48 ± 0.14
C10:0	7.27 ± 0.29	6.48 ± 0.39	7.23 ± 0.55
C11:0	0.13 ± 0.03	0.09 ± 0.02	0.09 ± 0.01
C12:0	4.72 ± 0.36	3.78 ± 0.39	4.69 ± 0.45
C13:0	0.09 ± 0.01	0.07 ± 0.02	0.10 ± 0.03
C14:0	12.15 ± 0.38	10.28 ± 0.49	11.30 ± 0.6
C14:1	0.24 ± 0.02	0.22 ± 0.08	0.21 ± 0.04
C15:0	1.22 ± 0.05^a^	0.91 ± 0.13^ab^	0.85 ± 0.06^b^
C15:1	0.17 ± 0.08	0.04 ± 0.00	0.10 ± 0.03
C16:0	33.16 ± 1.90	33.89 ± 0.49	38.19 ± 1.8
C16:1	1.31 ± 0.07	1.35 ± 0.26	1.18 ± 0.08
C17:0	0.93 ± 0.02^a^	0.81 ± 0.09^ab^	0.74 ± 0.02^b^
C17:1	0.29 ± 0.05^a^	0.31 ± 0.03^ab^	0.22 ± 0.22^b^
C18:0	7.01 ± 0.69	7.29 ± 1.13	6.32 ± 0.57
C18:1n-9c	20.71 ± 0.72^a^	22.80 ± 1.06^a^	18.17 ± 0.51^b^
C18:2n-6 t	0.12 ± 0.01	0.17 ± 0.01	0.14 ± 0.01
C18:2n-6c	1.96 ± 0.12^b^	2.39 ± 0.08^a^	1.93 ± 0.05^b^
C18:3n-3 r	0.22 ± 0.02^a^	0.12 ± 0.01^b^	0.10 ± 0.00^b^
C20:0	0.21 ± 0.01^a^	0.17 ± 0.01^b^	0.15 ± 0.01^b^
C20:4n-6	0.14 ± 0.01^b^	0.18 ± 0.01^a^	0.14 ± 0.01^b^
ΣSFA	68.39 ± 1.03	67.10 ± 1.35	73.34 ± 0.65
UFA	25.26 ± 0.94^a^	27.58 ± 1.36^a^	22.21 ± 0.60^b^
ΣMUFA	22.79 ± 0.83^a^	24.71 ± 1.29^a^	19.89 ± 0.58^b^
ΣPUFA	2.47 ± 0.13^b^	2.87 ± 0.10^a^	2.32 ± 0.05^b^

Values are means ±SE. Mean values without common superscript (a, b) differ significantly among LC, HS and HL groups (*P* < 0.05)

### Gene expression involved in milk fat production

**Fig. 2:**
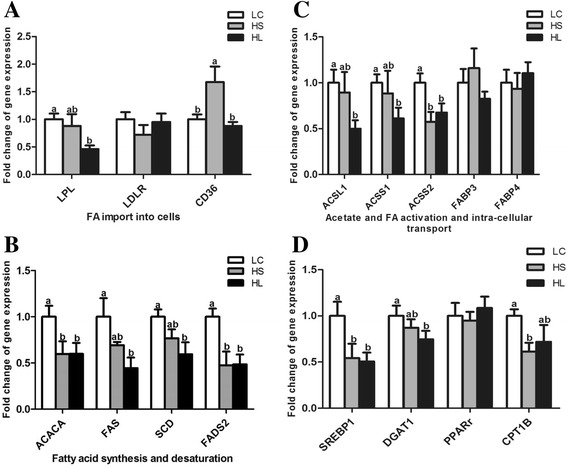
Genes expression involved in milk fat production in the mammary gland. **a** mRNA expression of genes involved in FAs up-take; **b** mRNA expression of genes regulating FAs synthesis and desaturation; **c** mRNA expression of genes for FAs activation and transport; **d** mRNA expression of genes involved in TG synthesis. Data are presented as mean ± SE. Mean values without common superscript (**a**, **b**) differ significantly among LC, HS and HL groups (*P* < 0.05)

As shown in Fig. 2, feeding a HC diet to lactating dairy goats down-regulated gene expression involved in milk fat production in the mammary gland. With respect to the fatty acid transport process, the mRNA expressions of the *LPL*, *ACSL1*, *ACSS1* and *ACSS2* genes were lower in the mammary gland of the HL goats (*P* < 0.05) and the CD36 and ACSS2 mRNA expressions were lower in the HS group compared with the LC group (*P* < 0.05). The expressions of the genes involved in fatty acids synthesis and desaturation including *SREBP-1*, *FAS*, *ACACA*, *SCD*, *FADS2,* and *DGAT1* were also down-regulated in the HL goats (*P* < 0.05) and the mRNA expressions of *ACACA, FADS2, SREBP-1,* and *CPT1B* were lower in the HS group compared with the LC group (*P* < 0.05). However, the levels of the *PPAR-γ*, *LDLR, FABP-3* and *FABP-* *4* mRNA expressions were not changed by feeding a HC diet (*P* > 0.05).

### Protein expression and FAS enzyme activity

**Fig. 3:**
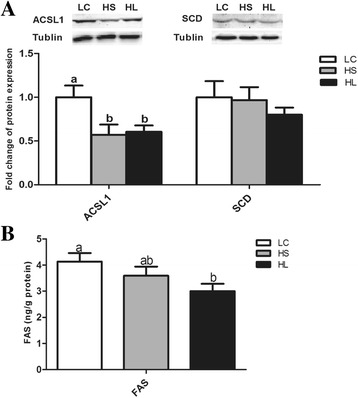
The level of the ACSL1 and SCD proteins expression and the FAS activity in the mammary gland. **a** Protein expression of ACSL1 and SCD; **b** FAS enzyme activity. Data are presented as mean ± SE. Mean values without common superscript (**a**, **b**) differ significantly among LC, HS and HL groups (*P* < 0.05)

The levels of the ACSL1 and SCD protein expressions in the mammary gland were determined by western blotting. The results showed that the level of ACSL1 protein expression in the mammary gland was decreased by feeding a HC diet (*P* < 0.05), while the SCD protein expression was not altered (*P* > 0.05). The activity of the FAS enzyme was also decreased in the HL goats compared with the LC group (*P* < 0.05) and there was no significant difference between the HS and LC groups (*P* > 0.05) (Fig. 3).

### DNA methylation analysis

**Fig. 4:**
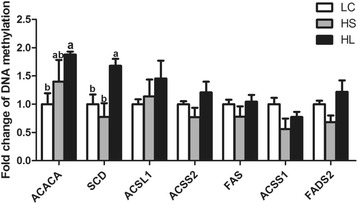
The level of the DNA Methylation in the promoter regions of the target genes. Data are presented as mean ± SE. Mean values without common superscript (**a**, **b**) differ significantly among LC, HS and HL groups (*P* < 0.05)

Due to a decrease in the gene expression involved in milk fat production in the mammary gland, the level of DNA methylation in the promoter regions of the encoding genes was measured using the MeDIP method. The results showed that the DNA methylation in the promoter regions of the *SCD* and *ACACA* genes was greater in the mammary gland of the HL goats compared with the LC group (*P* < 0.05). However, the levels of DNA methylation in the promoters of the *ACSL1*, *ACSS1* & *2*, *FAS* and *FADS2* genes were not changed in the HC goats compared with the LC group (*P* > 0.05) (Fig. 4).

## Discussion

To meet the energy demand of high milk production, dairy animals are commonly fed a HC diet, which likely causes abnormal fermentation in the rumen and leads to metabolic disorders known as SARA. Previous studies have shown that 19% of early lactation and 26% of mid-lactation cows experienced SARA [23]. Lactating animals suffering from SARA have increased risks for diarrhea, laminitis and inflammatory responses [24, 25]. This extensively-used feeding strategy ultimately leads to a decrease in milk quality with a lower quantity of milk fat [25–27]. In this study, as found in practice, our results showed that milk production was significantly increased by feeding a HC diet in both the HL and HS groups compared with the LC group, while the percentage of milk fat was significantly lower in the HL group. A previous study also demonstrated that feeding high grain diets to lactating dairy cows led to milk fat depression with lower milk fat concentrations [28].

Milk and dairy products contain fatty acids with high proportions of SFA and MUFA and small amounts of PUFA [29, 30]. Research has shown that humans consuming an excess of SFA have increased risk for coronary heart disease [31]. In contrast, UFAs benefit human health [4, 5]. Chilliard et al. observed that high-quality alfalfa hay-based diets can increase the level of C18:3n-3 in the milk of dairy cows [32]. However, cows fed a high starch diet experienced milk fat depression syndrome [28]. A low milk fat content has even been suggested as a noninvasive indicator to identify cows with a greater risk for SARA [33]. Previous studies have shown that lactating ruminants with SARA induced by feeding a HC diet did not experience altered concentrations of SFAs, PUFAs or MUFAs in the milk but had markedly decreased concentrations of C18:2n:6c, C18:3n3, and C20:3n6 [27, 34]. Our results showed that the concentrations of C18:1n-9c, SFAs and MUFAs were not significantly changed by feeding a short-term HC diet in the HS group as reported in a previous study [27]. However, it is very important to note that long-term feeding of a HC diet significantly decreased the percentage of milk fat and the concentrations of UFAs and MUFAs, while the concentration of SFAs exhibited an increasing tendency in the milk compared with the LC control goats.

Fatty acid synthesis is a complex process including uptake, transport, synthesis and oxidation of fatty acids. With respect to the FAs uptake, LPL is an important enzyme in the mammary gland responsible for taking up long-chain fatty acids from albumin-bound fatty acids, lipoproteins, or chylomicrons [35]. LPL also can hydrolyze triglyceride (TAG) in lipoprotein core and then deliver fatty acids to the mammary gland for milk fat synthesis [36]. The HL goats showed a significant decrease of the *LPL* mRNA expression in the mammary gland compared with the LC group as reported previously [34]. SREBP1 and PPAR γ are important transcription factors involved in milk fat synthesis [37, 38] and the lipogenic genes including *ACACA*, *FAS*, *ACSL1,* and *SCD* are all target genes of SREBP1 in bovine mammary epithelial cells [39, 40]. Our results showed that the *SREBP1* mRNA expression and its target downstream genes were significantly decreased in the mammary gland of the HS and HL groups compared with the LC group, while the *PPARγ* gene expression was not changed. Moreover, short- and medium-chain FAs (C4-C14), as well as some portions of the C16 FAs, are mainly synthesized de novo in the mammary gland [41]. ACACA is the rate-limiting enzyme involved in the FAs de novo synthesis by controlling the production of short chain fatty acid (SCFA) and palmitic from acetate [42]. Fatty acid synthase (FAS) is a multifunctional protein that can catalyze the majority of the enzymatic steps in the fatty acid synthesis [43]. In the present study, we found that, in conjunction with the decrease in total milk fat percent and some specific FAs concentrations including C15:0, C17:0, C17:1, C18:1n-9c, C20:0 and C18:3n-3r, the mRNA expressions of the *ACACA* and *FAS* genes and the enzyme activity of FAS were significantly downregulated in the mammary gland of the HL goats. Therefore, it is reasonable to speculate that the decrease in the gene expression and enzyme activity responsible for the FAs synthesis in the mammary gland may contribute to milk fat depression in HL goats.

As a desaturase, SCD is an important enzyme for controlling the intracellular FAs composition by catalyzing the conversion of SFA into monounsaturated FAs [44, 45]. In this study, we found that the *SCD* gene expression was significantly downregulated in the HL group, which may be responsible for the decrease in C18:1n-9c and the MUFA concentration in the milk of HL goats. Long-chain fatty acids were activated by acyl-CoA synthetase via long-chain family member isoforms (ACSL) [46], while short-chain fatty acids were activated by acyl-CoA synthetase short-chain family members (ACSS) [47]. ACSL1 is a predominant enzyme among the ACSL isoforms in the bovine mammary gland [48]. CD36 is another important enzyme controlling the long chain FAs uptake working in conjunction with intracellular fatty acid-binding proteins (FABP) [49, 50]. ACSS1 & 2 mainly regulate the oxidative pathway of lipids and acetate activation, respectively [47, 51]. In the present study, our results showed that the ACSL1 protein expression was also significantly decreased in the HL group compared with the LC group. The *ACSS2* mRNA expression was also markedly decreased in the HL goats. These results indicate a potential decrease in the milk fat synthesis in the mammary glands of HL goats compared with their LC counterparts.

Epigenetic modifications are involved in regulating gene transcription [52]. DNA methylation is perhaps the most extensively studied epigenetic modification and plays an important role in the regulation of gene expression [53]. For decades, methylation has been believed to play a crucial role in repressing gene expression through blocking the promoter region where the activating transcription factors should bind [54]. With respect to the general downregulation of the gene expression involved in the milk fat synthesis in the mammary gland, the status of the DNA methylation in the promoter regions of the target genes has been analyzed as previously described [51, 54]. The results demonstrated that the methylation of the *SCD* and *ACACA* promoters was significantly increased in the HL group compared with the LC group, which was consistent with the decrease in their gene expression. Consistently, previous studies also showed that the methylation in the *SCD* promoter region was increased in dairy goats and cows fed a high-concentration diet [54]. However, the methylation in the promoter regions of *FAS*, *ACSL1*, *ACSS1*, *ACSS2,* and *FADS2* DNA was not changed by feeding a HC diet. We speculate that the modification of the DNA methylation was involved in regulating the milk fat depression in the HL goats. In contrast, the DNA methylation in the lipogenic genes promoter was not significantly altered in the mammary gland of the HS goats compared to the LC group. To date, no information has been provided for explaining the difference in the DNA methylation between the HS and HL groups. Moreover, the mechanisms behind the changes in DNA methylation in the HL goats still require further investigation.

## Conclusions

Short-term feeding of a HC diet had minor effects on milk fat production and composition in lactating dairy goats. However, long-term feeding of a HC diet will induce milk fat depression and a FAs profile shift with lower MUFAs but higher SFAs. A downregulation of the gene expression involved in the process of lipid production and the upregulation of the DNA methylation in the mammary gland may contribute to the decrease in milk fat production in HL goats.

## Additional file

Additional file 1: Table S1. Ingredients and composition of the experimental diets (%). (DOCX 13 kb)

## Abbreviations

ACACA: Acetyl-coenzyme A carboxylase alpha
ACSL1: ﻿A﻿cyl-CoA synthetase long-chain family member 1
ACSS1: Acyl-CoA synthetase short-chain family member 1
ACSS2: Acyl-CoA synthetase short-chain family member 2
AGPAT: Acyl glycerol phosphate acyl transferase
CD36: Cluster of differentiation 36
CVD: Cardiovascular disease
DGAT: Diacylglycerol acyltransferase
DHA: Docosahexaenoic acid
EPA: Eico-sapentaenoic acid
FABP3: Fatty acids binding protein 3
FABP4: Fatty acids binding protein 4
FADS2: Fatty acid desaturase 2
FAS: Fatty acid synthase
GPAT: Glycerol-3 phosphate acyl transferase
LDLR: Low density lipoprotein receptor
LPL: Lipoprotein lipase
MUFA: Monounsaturated fatty acid
PPARγ: Peroxisome proliferator activated receptor gamma
PUFA: Polyunsaturated fatty acid
SARA: Subacute ruminal acidosis
SCD: Stearoyl-CoA desaturase
SCFA: Short chain fatty acid
SFA: Saturated fatty acid
UFA: Unsaturated fatty acid
